# Hot Deformation Behavior and Processing Map Considering Strengthening Effect for Al–10.0Zn–3.0Mg–2.8Cu Alloy

**DOI:** 10.3390/ma16051880

**Published:** 2023-02-24

**Authors:** Si-Qi Wang, Xi Zhao, Xian-Wei Ren, Zhi-Min Zhang, Xue-Dong Tian, Ya-Yun He

**Affiliations:** 1 School of Aerospace Engineering, North University of China, Taiyuan 030051, China; 2 Engineering Technology Research Center for Integrated Precision Forming of Shanxi Province, North University of China, Taiyuan 030051, China

**Keywords:** hot deformation, insoluble phase, processing map, strengthen, recrystallization

## Abstract

In this paper, a hot processing map that takes into the strengthening effect into account is optimized for the Al–10.0Zn–3.0Mg–2.8Cu alloy, mainly considering the crushing and dissolving behavior of the insoluble phase. The hot deformation experiments were performed by compression testing with strain rates ranging from 0.001 to 1 s^−1^ and the temperature ranging from 380 to 460 °C. The hot processing map was established at the strain of 0.9. It exhibits that the appropriate hot processing region is located at the temperature from 431 to 456 °C and its strain rate is within 0.004–0.108 s^−1^. The recrystallization mechanisms and insoluble phase evolution were demonstrated using the real-time EBSD-EDS detection technology for this alloy. It is verified that the work hardening can also be consumed by the coarse insoluble phase refinement with the strain rate increasing from 0.001 to 0.1 s^−1^, besides the traditional recovery and recrystallization, but the effect of the insoluble phase crushing was weakened when strain rate increased over 0.1 s^−1^. Better refinement of the insoluble phase was around strain rate in 0.1 s^−1^, which exhibits adequate dissolving during the solid solution treatment, leading to excellent aging strengthen effects. Finally, the hot processing region was further optimized, so that the strain rate approaches 0.1 s^−1^ instead of 0.004–0.108 s^−1^. This will provide a theoretical support for the subsequent deformation of the Al–10.0Zn–3.0Mg–2.8Cu alloy and its’ engineering application in aerospace, defense and military fields.

## 1. Introduction

High mechanical properties, low cost, and lightweight components attract attention in the aerospace and transportation fields [1,2,3]. Al–Zn–Mg–Cu alloy has the advantages of high specific strength, high specific stiffness and favorable ductility. It is the first choice for lightweight equipment [4,5]. However, in recent years, with the development of modern industry, the demand for lightweight, high strength-ductility and low energy consumption structural materials is increasing. The service conditions of structural materials in national defense and aerospace fields are becoming more and more demanding, and products with better performance are needed to cope with practical use. Scholars have improved the strength of Al–Zn–Mg–Cu alloy by increasing the degree of alloying. With increasing content of Zn and Mg elements for the Al–Zn–Mg–Cu alloys, numerous nano-scale phases precipitate (MgZn phase) after deformation and aging treatment, resulting in the enhancement of the mechanical properties [6,7]. However, it is prone to hot cracking for the Al–Zn–Mg–Cu ingot when the Zn element exceeds 8.5 wt.%, especially for ingots with diameters greater than 100 mm [8]. In this study, the Al–10.0Zn–3.0Mg–2.8Cu alloy ingot in 260 mm diameter was processed using the semicontinuous casting process coupled with an electromagnetic field. Homogeneous, high-purity and high-alloyed Al–10.0Zn–3.0Mg–2.8Cu alloy is obtained by this cast technology, and the increase of key alloying elements (Mg, Zn) can produce higher precipitate volume fraction. After plastic deformation and heat treatment, the alloy has the potential of 800 MPa. This strongly promotes the replacement of medium-strength steel with aluminum alloys, showing excellent engineering application value. As a new material, its hot processing parameters are unknown, and it is of great significance to study the hot deformation behavior and processing map of the new Al–10.0Zn–3.0Mg–2.8Cu alloy.

The construction of the hot processing map can determine the hot deformation conditions of alloys and show safe processing regions or flow instability processing regions [9]. Researchers usually use the power dissipation (η) value combined with microstructure analysis of the characteristic area to determine the optimal processing parameters. Bin Ke et al. [10] researched the hot deformation behavior of AA7020 within 673–793 K/0.001–1 s^−1^ and established 3D processing map. It concluded that 743–793 K/0.004–0.05 s^−1^ are the domain suitable for hot working. Shi et al. [11] established the hot processing map of high alloy 7000 series alloy. It found that dynamic recovery (DRV), continuous dynamic recrystallization (CDRX) and discontinuous dynamic recrystallization (DDRX) participate the alloy softening. Zhao et al. [12] found that DRX played an important role in the Al–Zn–Mg–Cu alloy during the hot compression at the temperatures of 300–450 °C within the low strain rate range 10^−5^–10^−3^ s^−1^. The insoluble phase (AlZnMgCu particle) for Al–Zn–Mg–Cu alloys with high zinc content (10 wt.%) increased with the increase of Zn content [13]. These coarse particles can be crushed during the deformation, assisting the deformation energy dissipation [14], and the coarse phase (>1 μm) also promote particle stimulated nucleation (PSN) during hot deformation [15]. Therefore, for Al–Zn–Mg–Cu alloy with high Zn content, the effect of the insoluble phase on the hot deformation should be considered.

In recent years, a large number of studies have reported that the coarse particles of Al–Zn–Mg–Cu alloy has an important effect on the hot deformation behavior. R. K. Gupta et al. [16] carried out hot isothermal compression test on AA7010 and AA7075 and determined the hot working safety region of the two alloys. The results show that the second phase can be used as the nucleation point of DRX, which has an important effect on controlling the hot deformation behavior of the alloy. Zang et al. [17] studied that the particles act as nucleation sites for the Al–7.9Zn–2.7Mg–2.0Cu alloy, which induced the PSN at low deformation temperature. Cheng et al. [14] found that phase particles experience fragmentation during ECAP at 473 K and these particles effectively coordinated deformation. Meanwhile, the crushed second phase particles are easier to dissolve in the subsequent solid solution treatment to achieve a higher saturation degree, which will facilitate the dispersive precipitation after aging treatment and effectively enhance the strength-ductility of the alloy [18,19]. However, for Al–Zn–Mg–Cu with high Zn content, the influence of temperature and strain rate on the hot deformation behavior is usually considered in the existing hot processing maps, but there are few studies about the influence of the insoluble particles on hot deformation behavior coupled with subsequent solution-aging strengthening effects for Al–Zn–Mg–Cu alloy.

In this work, the hot deformation behavior and processing map considering the strengthening effect of the Al–10.0Zn–3.0Mg–2.8Cu alloy were investigated from three aspects. First, the microstructure characteristics and recrystallization mechanisms were determined by the real-time EBSD-EDS characterization technology. Second, the evolution law of the insoluble phase was obtained and the hot processing map was further optimized. Third, the optimal processing area was verified by subsequent solution-aging treatment and hardness tests. The processing region considering the strengthening effect of this alloy was proposed eventually, which opens up a new research idea to find the hot deformation processing for such alloys.

## 2. Materials and Methods

**Figure 1:**
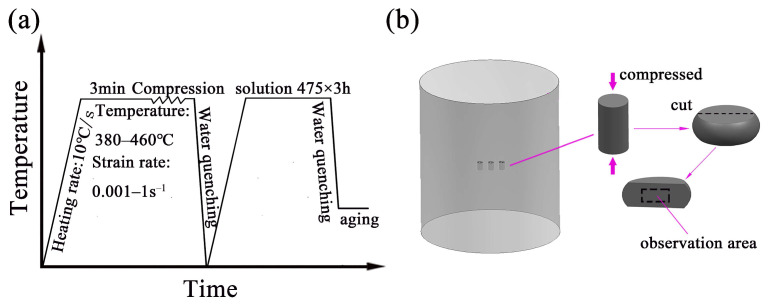
(**a**) Processing diagram, (**b**) Sampling scheme.

In this study, a new Al–Zn–Mg–Cu alloy of Central South University was used, which was homogenized at 470 °C × 20 h. Its chemical compositions are shown in Table 1. Hot compression experiments were carried out on a Gleeble-3500 thermo-simulation machine. According to related research, the experimental parameters of hot compression of Al–Zn–Mg–Cu alloy are usually in the range of 300–500 °C/0.001–10 s^−1^ [10,11,12,16,17]. Combined with the actual experimental conditions of subsequent plastic deformation of Al–10.0Zn–3.0Mg–2.8Cu alloy large components, the selection of deformation parameters is 380–460 °C, and strain rates range from 0.001 to 1 s^−1^. The cylindrical samples of 8mm diameter and 12 mm height were processed. Figure 1 shows the processing diagram and sampling scheme.

**Figure 2:**
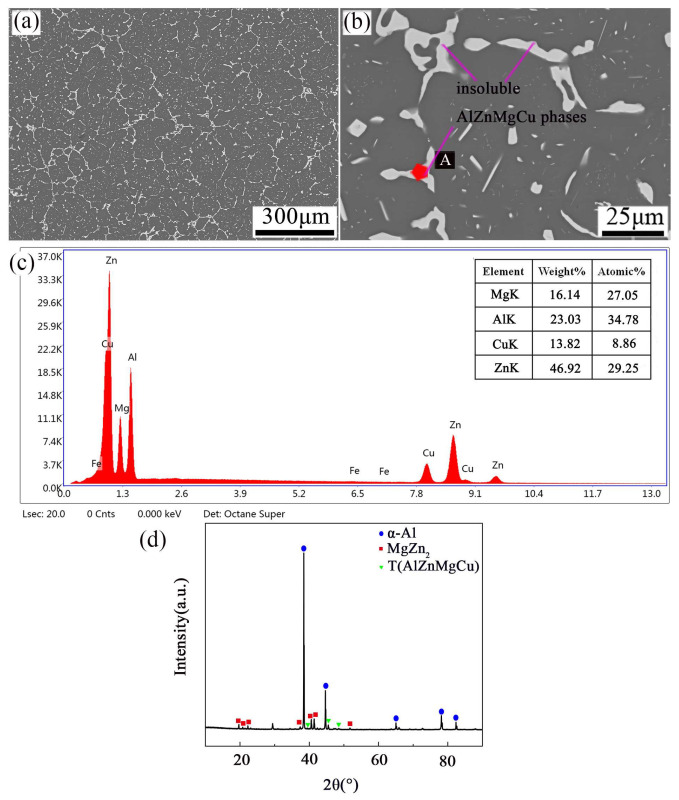
Initial microstructure of Al–10.0Zn–3.0Mg–2.8Cu alloy. (**a**) overall microstructure (**b**) coarse phase (**c**) EDS result of point A (**d**) XRD result.

The initial microstructure consists of coarse equiaxed grains, whose average size is about 180 μm (Figure 2a). The coarse grain boundary phase in Figure 2b was scanned by EDS (SU5000, Hitachi, Tokyo, Japan), and the element distribution of point A in Figure 2b was measured as shown in Figure 2c. This coarse phase is an insoluble Cu-rich AlZnMgCu(T) phase. It can become a crack source when stress is applied, causing damage to the mechanical properties [20], and the fine rod phase is uniformly dispersed within α(Al) and belongs to MgZn_2_(η or η′) [21]. The XRD diffraction pattern of the homogenized alloy is shown in Figure 2d. It can be seen that, in addition to α-Al, there are mainly MgZn_2_ phase and T phase in the initial structure, which is consistent with the SEM analysis results.

After compression the microstructure located in the central section was observed using a scanning electron microscope (SEM, SU5000, Hitachi, Tokyo, Japan), electron backscattered diffraction (EBSD, SU5000, Hitachi, Tokyo, Japan), and energy dispersive spectrometer (EDS, SU5000, Hitachi, Tokyo, Japan). Real-time EBSD–EDS detection technology was adopted to scan the same area to obtain inverse pole figures (IPF) and EDS maps synchronously. EBSD was conducted at 20 kV, 70° tilt angle, and the OIMv7.3 software was used to analyze data. Low angle grain boundaries (LAGB, misorientation angle of 2–15°) and high angle grain boundaries (HAGB, misorientation angle over 15°) were marked using thin white lines and thick black lines, respectively. Thin red lines in the EDS map represent LAGB. In addition, to obtain reliable second phase particle statistical results, three SEM images of different magnifications were collected for each sample under different conditions. The hardness was tested by THBP62.5TIME Brinell (Beijing TIME High Technology Ltd., Beijing, China) hardness tester. The specimens were mechanically polished before the test, and the average value of 10 tests at different positions was calculated. The diameter of the indenter used was 2.5 mm, and the spacing between each test point was no less than 3 mm.

## 3. Hot Deformation Behavior

### 3.1. Constitutive Model

**Figure 3:**
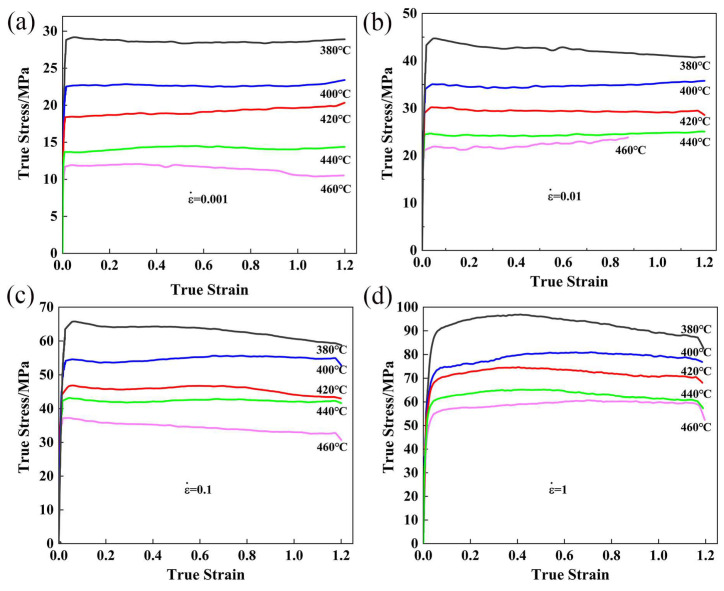
The curves of true stress–true strain curves of Al–10.0Zn–3.0Mg–2.8Cu alloy (**a**) 0.001 s^−1^, (**b**) 0.01 s^−1^, (**c**) 0.1 s^−1^, and (**d**) 1 s^−1^.

The flow stress-true graphs are shown in Figure 3. The influence of deformation condition on the flow stress are remarkable. As the temperature drops and the strain rate improves, the true stress increases. Flow stress increases rapidly in the initial stage and reaches peaks, then remains constant or decreases slightly finally. It means that dynamic softening competes with work hardening and achieved dynamic balance.

To clarify the relationship between the flow stress and deformation parameters, constitutive models were established using the Arrhenius equation [22]. The mathematical expressions among temperature, strain rate, and stress are expressed using Equation (1).

(1) $$\dot{\varepsilon }=A{\left[\sinh\left(\alpha \sigma \right)\right]}^{n}\exp\left(Q/\mathrm{RT}\right)$$

In the formula, A_1_, A_2,_ A, n_1_, n, α and β are constant, $\dot{\varepsilon }$ is the strain rate, σ is the flow stress, T and R are the absolute temperature (K) and gas constant (8.314 J/(mol·K)) respectively and Q is the activation energy.

The Zener–Hollomon parameter represents the comprehensive effect of deformation temperature and strain rate on the deformation [23]:

(2) $$Z=A{\left[\sinh\left(\alpha \sigma \right)\right]}^{n}=\dot{\varepsilon }\exp\left(Q/\mathrm{RT}\right)$$

The Arrhenius constitutive equation was set up using the Z parameter:

(3) $$\sigma =1/\alpha \ln\left\{{\left(Z/A\right)}^{1/n}+{\left[{\left(Z/A\right)}^{1/n}+1\right]}^{1/2}\right\}$$

**Figure 4:**
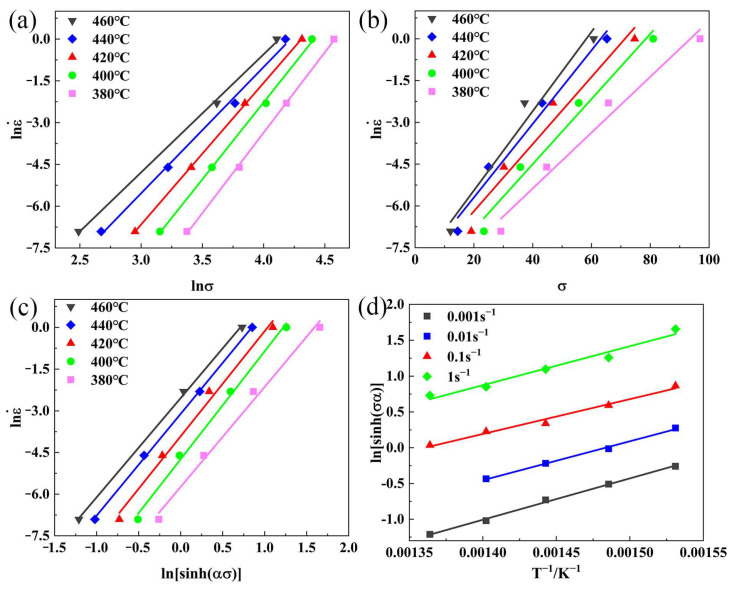
Relationship between (**a**) $\ln\dot{\varepsilon }-\ln\sigma$, (**b**) $\ln\dot{\varepsilon }-\sigma$, (**c**) $\ln\dot{\varepsilon }-\ln\left[\sinh\left(\alpha \sigma \right)\right]$, (**d**) $\ln\left[\sinh\left(\sigma \alpha \right)\right]-1/T$.

Figure 4 shows the fit curves $\ln\dot{\varepsilon }-\ln\sigma$,$\;\ln\dot{\varepsilon }-\sigma$, $\ln\left[\sinh\left(\sigma \alpha \right)\right]-\ln\dot{\varepsilon }$, $\ln\left[\sinh\left(\sigma \alpha \right)\right]-1/T$ at different temperatures. Table 2 gives the parameters from the constitutive equation. Combined with Equation (3), the hot deformation behavior of new Al–10.0Zn–3.0Mg–2.8Cu alloy can be expressed by the Z parameter in Equation (4), and the equation of the Z parameter is given in Equation (5).

(4) $$\sigma =41.31\ln\left\{{\left(Z/2.38\times 10^{10}\right)}^{1/3.72}+{\left[{\left(Z/2.38\times 10^{10}\right)}^{2/3.72}+1\right]}^{1/2}\right\}$$

(5) $$Z=\dot{\varepsilon }\cdot \exp\left(160,427/\mathrm{RT}\right)$$

**Figure 5:**
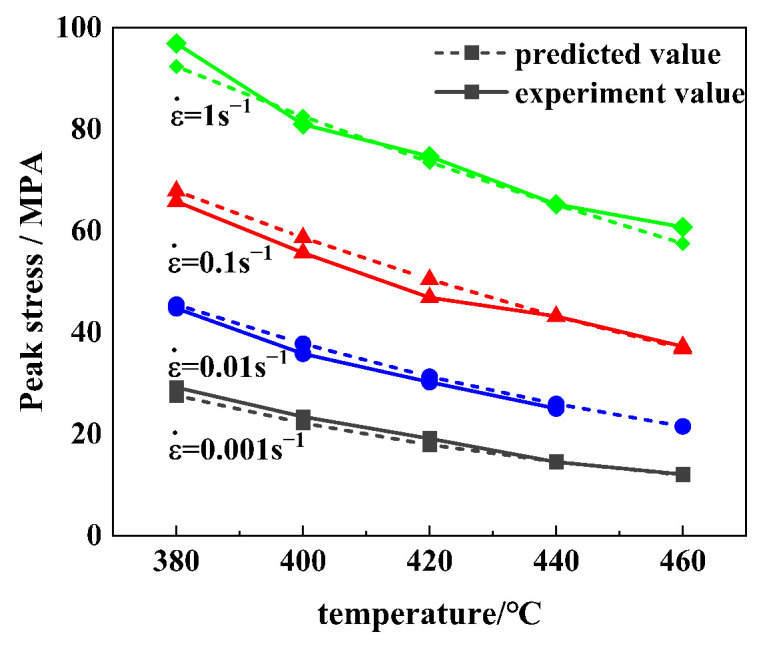
Comparison of peak stress between experiment value and predicted value.

To evaluate the accuracy and reliability of the constitutive equation on hot deformation behavior, the peak stress test data were compared with the predictive value using the Arrhenius models, as shown in Figure 5. The predictive value is consistent with the experimental data, and the prediction accuracy of peak stress can reach 0.966, explaining that the established constitutive model has a preferable prediction power.

### 3.2. Hot Processing Map

The dynamic material model (DMM) is commonly used to construct the processing map of the aluminum alloys [24]. This model combines the external energy with the energy consumed during the hot deformation process.

When the material is deformed, the energy required for microstructure evolution is usually expressed by power dissipation efficiency η, which is defined as follows [25]:

(6) $$\eta =J/J_{\max}=2m/\left(1+m\right)$$

The flow instability criterion for Kumar–Prasad (K–P) is as follows [26]:

(7) $$\xi \left(\dot{\varepsilon }\right)=\partial \ln\left[m/\left(1+m\right)\right]/\partial \ln\dot{\varepsilon }+m<0$$

**Figure 6:**
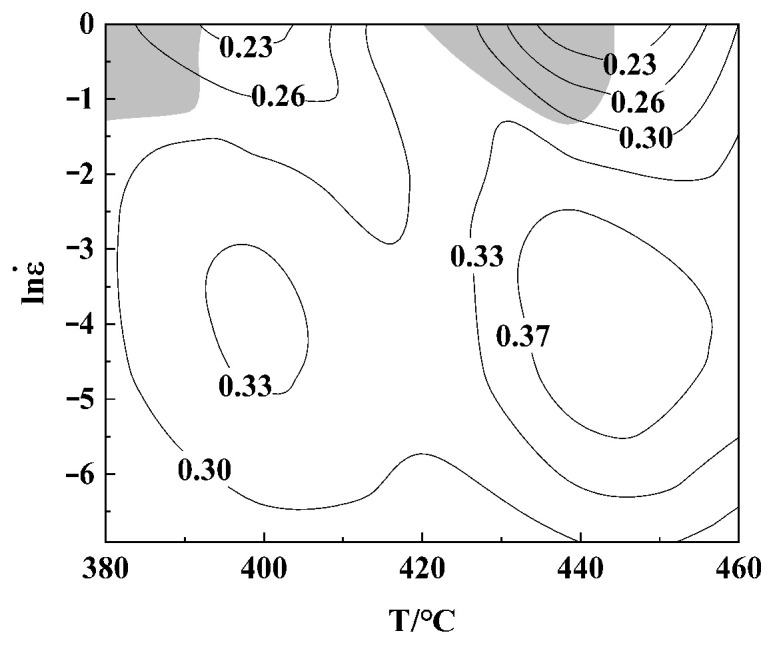
Processing map of the Al−Zn−Mg−Cu alloy at 0.9 strain.

Based on Equations (6) and (7), the hot processing map of the investigated alloy at 0.9 strain is established. The shaded area is the flow instability zone (Figure 6), and the rest is the safe zone. The energy dissipation coefficient changing trend is shown in the contour map. The change in the η contour line denotes the evolution of specific microstructure under certain deformation condition. Therefore, the η contour is called the ‘microstructure trajectory’ [27]. The peak value of the power dissipation factor η is 37%. The appropriate processing region may exist within the temperature range of 431–456 °C, and the strain rate range is 0.004–0.108 s^−1^.

## 4. Results and Discussion

According to the obtained process parameter range, the deformation parameter was set as 440 °C/0.001–1 s^−1^. The microstructure characteristics and insoluble phase evolution were investigated. In addition, as the deformation progresses, the billet temperature will decrease. Therefore, the low deformation temperature (380 °C) with the different strain rates was studied. Furthermore, the effect of the insoluble phase on strengthening under the subsequent solution-aging process was demonstrated.

### 4.1. Microstructure Characteristics within Hot Processing Map

Softening and work hardening mechanisms of materials compete with each other in the deform process. DRV has a major impact on the softening mechanism of aluminum alloys because it has high stacking fault energy. Meanwhile, it was reported that the softening effect caused by DRX during hot processing is also important [28]. The slow strain rate provides sufficient time for the microstructure evolution during compression. Therefore, the microstructure characteristics and recrystallization mechanisms at 380 °C/0.001 s^−1^ and 440 °C/0.001 s^−1^ were investigated.

**Figure 7:**
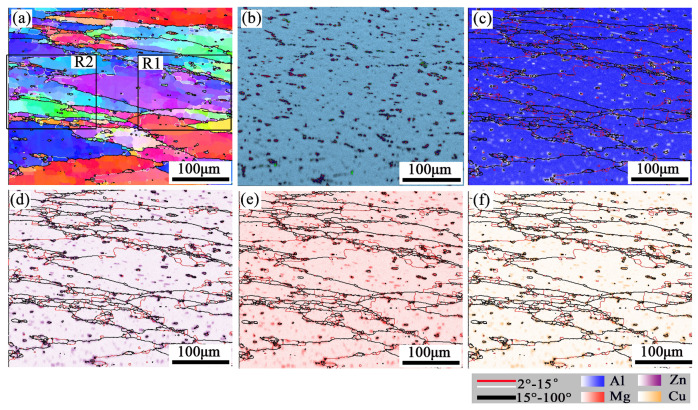
(**a**) EBSD-IPF map (**b**) EDS map and energy spectrum analysis maps: (**c**) Al, (**d**) Zn, (**e**) Mg, (**f**) Cu at 380 °C/0.001 s^−1^.

**Figure 8:**
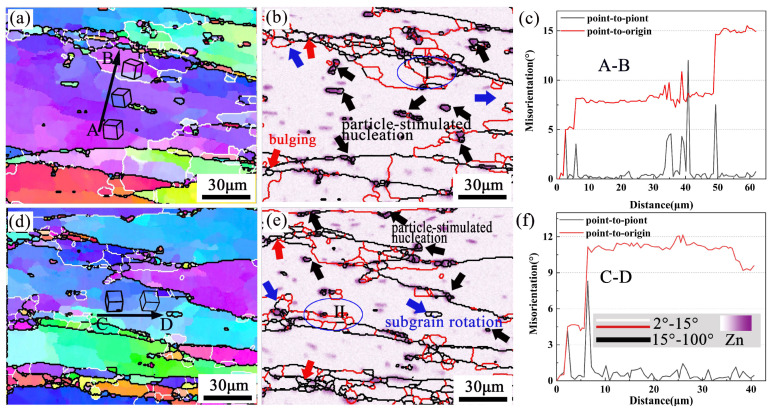
The typical region R1 (**a**–**c**) and R2 (**d**–**f**) selected in EBSD -EDS maps partially enlarged at 380 °C/0.001 s^−1^.

The results of the real-time EBSD-EDS microstructure for the sample (380 °C/0.001 s^−1^) are shown in Figure 7 and Figure 8. The grains are elongated in the direction perpendicular to the compression direction, and slightly fine DRXed grains are formed in intracrystalline and grain boundaries. The DRXed grains are divided into three categories. Firstly, some recrystallized grains were formed through AlZnMgCu particle stimulation, located in the intragranular and grain boundary. Secondly, some grains are formed near the serrated grain boundary through grain boundary bulging. Thirdly, some recrystallized grains can be promoted through subgrain rotation, and this mode of recrystallization yields few grains.

Figure 8 is a partially enlarged detail of typical regions (R1 and R2), in the black rectangle of Figure 7. Figure 8b,e are the EDS maps of the Zn element. DRX occurs around the second phase particles which are rich in Zn, as indicated by the black arrows. Because of the strain gradient caused by second phase particles during deformation, the nucleation growth of small recrystallized grains is promoted, this belongs to DDRX [29]. Moreover, many fine recrystallized grains were distributed at the zigzag grain boundaries (as red arrows in Figure 8b,e). The grain boundaries were driven toward high-energy storage and generated new recrystallized nuclei because of the strain gradient between adjacent grains during compression. The black line A→B/C→D (Figure 8a,d) represents the misorientation distribution from point to point along the arrow. The grain color changes on the line, indicating that the dislocation movement ability in this area is high. The point-to-point line was above 0° and the point-to-origin line exceeded 10° representing subgrain rotation (Figure 8c,f). Rotation of the crystal lattice leads to the LAGBs transform into HAGBs, thus forming new grains (as marked by blue arrows). It represents a typical CDRX occurrence [30]. Many subgrains are observed in the original grain, shown as Ⅰ and Ⅱ in Figure 8b,e. This may be attributed to the inadequacy of CDRX or sub-dynamic recrystallization. If hot deformation continues, the LAGBs will absorb the dislocations and transform into the HAGBs, forming CDRX grains [31].

**Figure 9:**
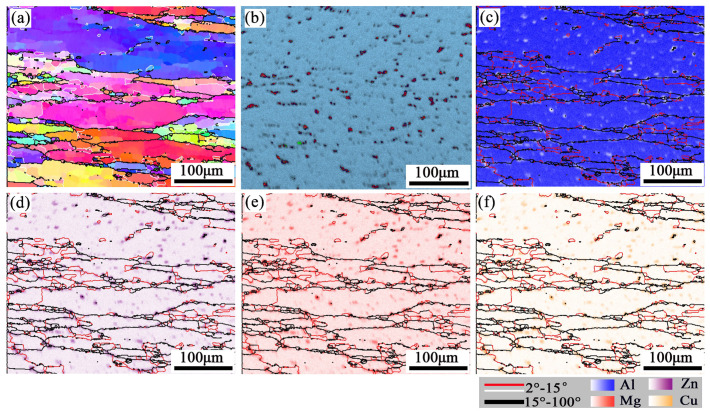
(**a**) EBSD-IPF map (**b**) EDS map and energy spectrum analysis maps: (**c**) Al, (**d**) Zn, (**e**) Mg, (**f**) Cu at 440 °C/0.001 s^−1^.

**Figure 10:**
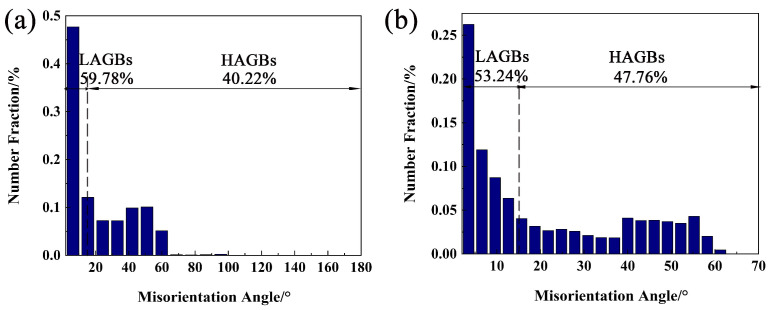
Misorientation Angle (°): (**a**) 380 °C/0.001 s^−1^, (**b**) 440 °C/0.001 s^−1^.

A similar phenomenon can be discovered in the deformed microstructure at 440 °C/0.001 s^−1^ (Figure 9). The microstructure consists of extruded elongated grains and some fine recrystallized grains. In addition, the percentage of LAGBs in the elongated large grains decreases but the HAGBs increase. The histogram of misorientation angle is shown in Figure 10. The pictures show that the LAGBs tend to transform into HAGBs, and the subgrains are gradually transformed into recrystallized grains. The recrystallized grains formed through AlZnMgCu particles are less than 380 °C/0.001 s^−1^ (Figure 7 and Figure 9). That is because some large insoluble particles dissolved during the deformation temperature elevate, and the DDRX caused by the PSN was weakened at 440 °C/0.001 s^−1^.

In summary, the recrystallization mechanism mainly includes the DDRX which comes from PSN, grain boundary bulging, and CDRX. Meanwhile, inadequate CDRX can be found surrounded by HAGBs and LAGBs. After hot pressing at 380 °C/0.001 s^−1^, the second phase particles are retained, which promoted the recrystallization of PSN during hot deformation. However, for the alloy after hot compression at 440 °C/0.001 s^−1^, the PSN weakened as the compression temperature rises to 440 °C. Compared the hot deformation activation energy (Q) of new Al–10.0Zn–3.0Mg–2.8Cu alloy (Q,160.43 KJ/mol) with other research results, such as 7075 (246.16 KJ/mol) [32], Al–5Mg–3Zn–1Cu (183.49 KJ/mol) [33], 7150 (229.75 KJ/mol) [34], AA7085 (249.11 KJ/mol) [35]. This alloy has lower activation energy which means a better hot deformation ability.

### 4.2. Optimization of Hot Processing Map Considering the Insoluble Phase

**Figure 11:**
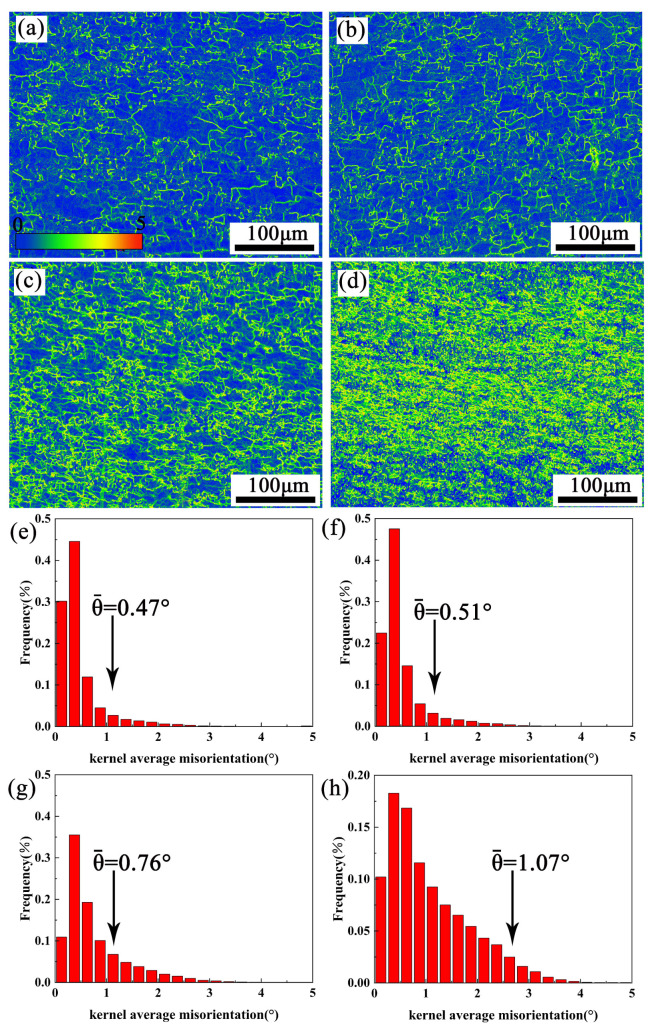
EBSD-kernel average misorientation (KAM) maps and local misorientation average angle of partial recrystallization for samples at 380 °C: (**a**,**e**) 0.001 s^−1^, (**b**,**f**) 0.01 s^−1^, (**c**,**g**) 0.1 s^−1^ (**d**,**h**) 1 s^−1^.

**Figure 12:**
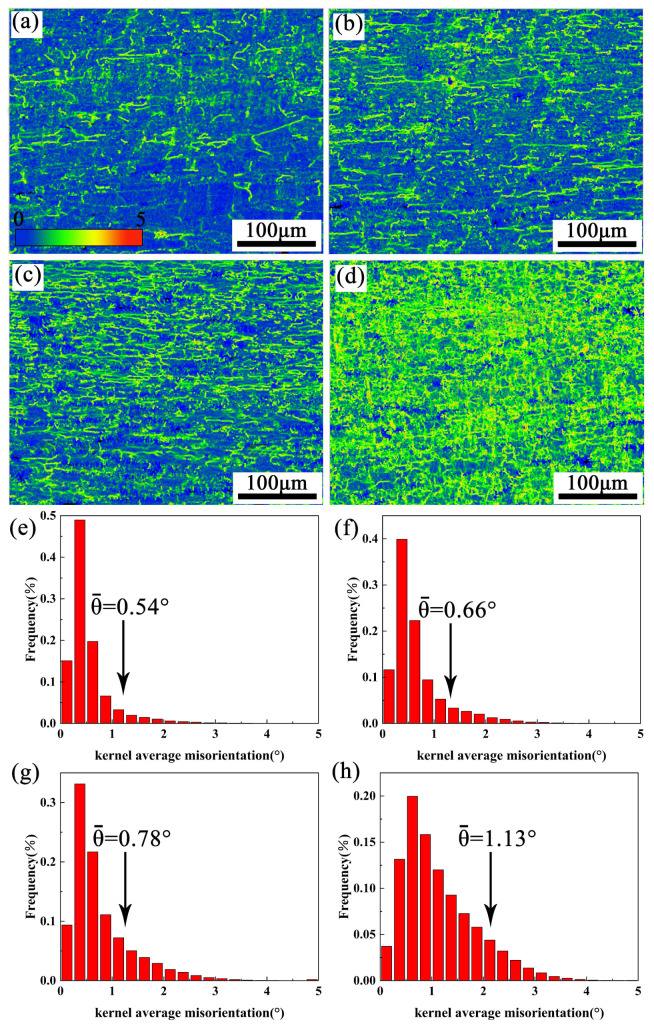
EBSD-kernel average misorientation (KAM) maps and local misorientation average angle of partial recrystallization for samples at 440 °C: (**a**,**e**) 0.001 s^−1^, (**b**,**f**) 0.01 s^−1^, (**c**,**g**) 0.1 s^−1^, (**d**,**h**) 1 s^−1^.

Figure 11 and Figure 12 show the Kernel average misorientation (KAM) distributions of the alloys at 380 °C/0.001–1 s^−1^ and 440 °C/0.001–1 s^−1^. The KAM value from 0 to 5° corresponds to the change from blue to red. As the strain rate rises from 0.001 to 1 s^−1^, the overall color of the image changes from blue to green and then to yellow. This distribution indicates that the degree of dislocation accumulation deepens with the increase of the strain rate. The average value of KAM at 380 °C and 440 °C under 0.001–1 s^−1^ is in the corresponding KAM distribution histogram, as shown in Figure 11e–h and Figure 12e–h. The dislocation density (ρ) can be calculated through KAM value [36,37,38,39].

(8) $$\rho =2\theta /\left(\mu b\right)$$

where θ represents the local misorientation profile in the KAM map, μ represents the scanning step of EBSD (0.65 μm), and b is the Burgers vector (0.286 nm). The ρ values are listed in Table 3. It shows that the ρ increases with the strain rate from 0.001 to 1 s^−1^. This is because there is insufficient time for the dislocations to move at high strain rates, resulting in a large amount of dislocation accumulation.

**Figure 13:**
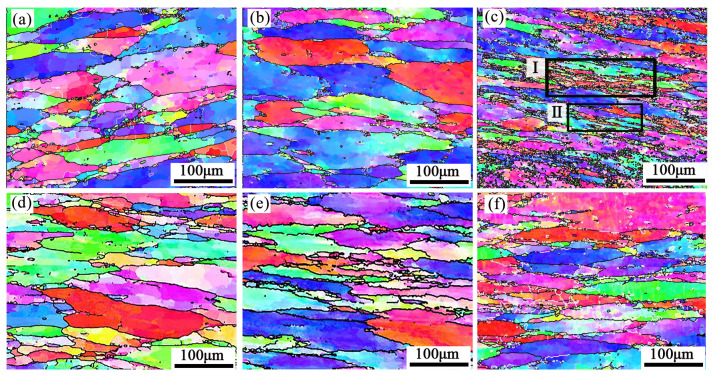
EBSD-IPF maps of the specimens deformed at 380°C: (**a**) 0.01 s^−1^, (**b**) 0.1 s^−1^, (**c**) 1 s^−1^ and deformed at 440 °C: (**d**) 0.01 s^−1^ (**e**) 0.1 s^−1^, (**f**) 1 s^−1^.

**Figure 14:**
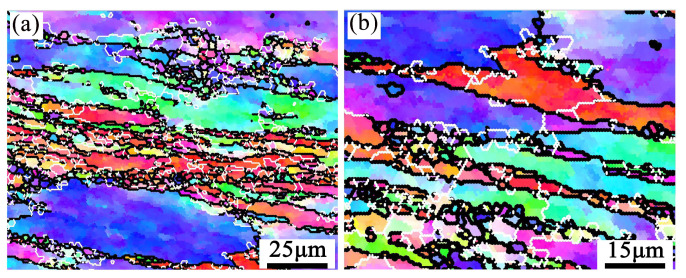
The enlarged images of black rectangle in Figure 13c. (**a**) Ⅰ,(**b**) Ⅱ.

The microstructure evolution of the deformed samples at 380 and 440 °C under 0.01–1 s^−1^ is shown in Figure 13. There mainly conclude elongated deformed grains and some fine recrystallized grains. The shear bands are shown as the zone in the black rectangle Ⅰ and black rectangle Ⅱ (Figure 13c), and their enlarge images show in Figure 14. According to the results of Section 3.2, the microstructure features verified the instability areas in the hot processing map. Due to the large density dislocation accumulation, the shear band becomes the favorable nucleation position for DDRX [40]. Therefore, many fine recrystallized grains occur in Figure 13c. The DRX fraction (f_DRX_) statistical results are in Table 4. It reveals that the f_DRX_ increased with the strain rate rising to 1 s^−1^. This is because a large number of dislocations pile up at a higher strain rate. Then the more energy accumulated, the easier recrystallization nucleated.

**Figure 15:**
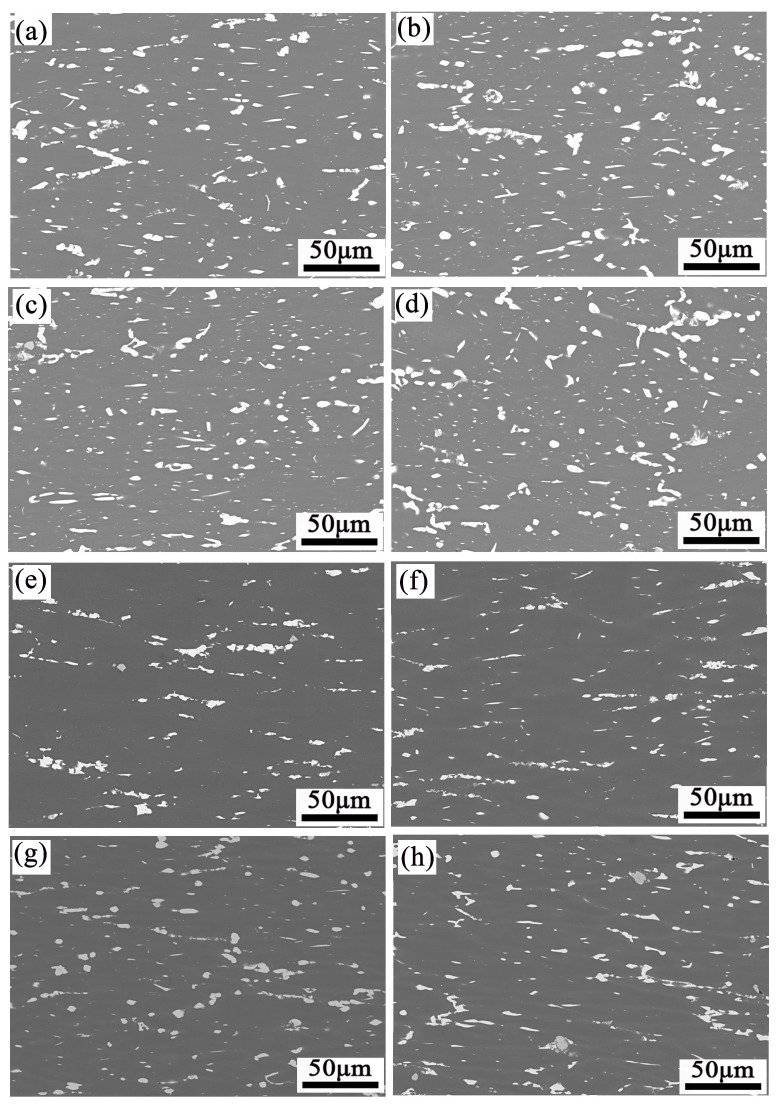
BSE maps of specimens deformed in different conditions: (**a**) 380 °C/0.001 s^−1^, (**b**) 380 °C/0.01 s^−1^, (**c**) 380 °C/0.1 s^−1^, (**d**) 380 °C/1 s^−1^, (**e**) 440 °C/0.001 s^−1^, (**f**) 440 °C/0.01 s^−1^, (**g**) 440 °C/0.1 s^−1^, (**h**) 440 °C/1 s^−1^.

**Figure 16:**
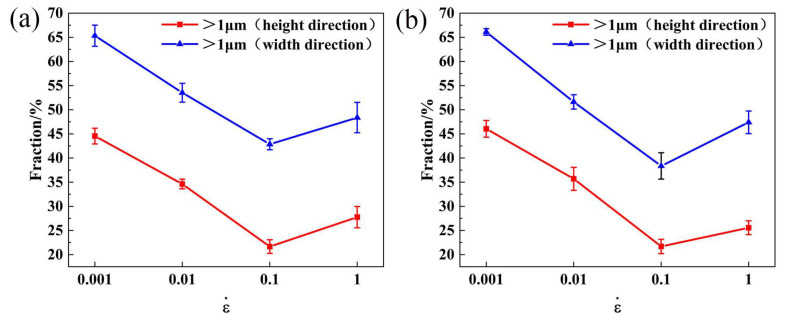
Statistics of phases in samples with different deformation conditions: (**a**) 380 °C, (**b**) 440 °C.

The backscattered electron (BSE) micrographs of the samples at 380 and 440 °C under different strain rates show in Figure 15. As the deformation temperature elevates to 440 °C, the number of coarse insoluble particles drops obviously because the temperature increase can promote the dissolution of particles. To quantitatively analyze the degree of fragmentation of the second phase, the proportion of large particles (>1 μm) in the total particles was counted. Its results are shown in Figure 16. The fraction of particles (>1 μm) decreased as the strain rate increased to 0.1 s^−1^. The reason is that the high strain rate leads to a large dislocation density, and it refines the coarse insoluble phase. The mechanical cleavage of particles is positively correlated with the dislocation density [41]. It means that the crushing of the coarse insoluble phase consumes work hardening during the hot deformation process and promotes the softening of the alloy. As the strain rate rises from 0.1 to 1 s^−1^, the percentage of particles (>1 μm) rises again, and the particle refinement is restrained. The reason may be that the greater f_DRX_ consumes more deformation energy, which weakens the refinement of the insoluble phase at this time (Figure 13 and Table 4). In conclusion, whether at 380 or 440 °C, the crushing effect of the insoluble phase is the best in 0.1 s^−1^ strain rate.

Combining the second phase particle breakage, dislocation density evolution and recrystallization behavior, and discussed as follows. The ρ values increased with the strain rate, enhancing yield stress or work hardening. The activation of the recrystallization and particle breakage regulates the stress reaching equilibrium together. Particles participate in dislocation consumption as the strain rate increases to 0.1 s^−1^. They were broken during deformation. Meanwhile, the proportion of recrystallized grains increased. It is verified that the PSN was weakened, accompanied by the enhancement of other recrystallization mechanisms. However, when the strain rate increases to 1 s^−1^, a shear band is observed at 380 °C and particle refinement was inhibited. These phenomena promote the development of recrystallization. With the deformation temperature increased to 440 °C, the shear band disappears, DDRX by PSN and grain boundaries bulging still exist. Thus, it is observed that recrystallization initiates softening first, rather than cutting the second phase particles when plenty of dislocations are applied to the matrix. In summary, when the strain rate is 0.1 s^−1^ (380 and 440 °C), the insoluble phase is sufficiently broken, which can avoid stress concentration and cracks during processing. At the same time, the degree of recrystallization is better, which can consume work hardening with broken second phase particles together, so that the plastic deformation of the alloy can be well coordinated.

Based on the recommended processing areas (431~456 °C, 0.004~0.108 s^−1^) in the hot processing map in Section 3.2, the strain rate can be further optimized to be close to 0.1 s^−1^ according to the evolution law of the insoluble phase. The fine insoluble phase will increase the degree of the dissolving of the solid solution and then obtain a better strengthening effect. Next, relevant experiments are carried out to verify this conclusion.

### 4.3. Verification of Optimal Hot Processing Region

**Figure 17:**
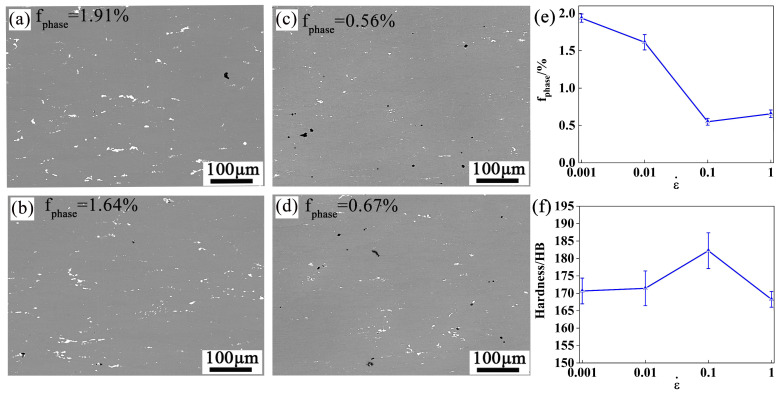
BSE maps of specimens deformed in different conditions after heat treatment: (**a**) 440 °C/0.001 s^−1^, (**b**) 440 °C/0.01 s^−1^, (**c**) 440 °C/0.1 s^−1^, (**d**) 440 °C/1 s^−1^ and (**e**) Statistics of fphases%, (**f**) Hardness distribution.

To verify the accuracy of the appropriate hot processing region, the sample was treated with a solution treatment at 440 °C for 475 × 3 h. The dissolution of the insoluble phase was observed. After solution treatment, the peak aging was carried out at 120 °C for 24 h, and the microhardness was tested. Figure 17 shows the SEM images at 440 °C/0.001–1 s^−1^, the statistical data of insoluble phase area fraction and the hardness distribution. Figure 17a–d show that the dissolution degree of 440 °C/0.1 s^−1^ is significantly larger than other deformation conditions. As the strain rate rises from 0.001 s^−1^ to 0.1 s^−1^, the percentage of the coarse insoluble phase decreases from 1.91 to 0.56% (Figure 17e). The percentage of the insoluble phase is 0.56% at 0.1 s^−1^ strain rate, it reached the minimum value. As the strain rate exceeds 0.1 s^−1^, the fraction of the coarse insoluble phase increases slightly. This is consistent with the finer insoluble phase makes the higher degree of dissolving in Section 4.2.

The hardness distribution of the peak-aged specimens shows opposite rules with the fraction of the insoluble phase, as shown in Figure 17f. When the strain rate rises from 0.001 s^−1^ to 0.1 s^−1^, the hardness enhances from 171.7 HB to 174.9 HB. However, when the strain rate rises to 1 s^−1^, the hardness decreased to 168.6 HB. The hardness of the alloy can be used as a reference value of its yield strength to some extent [42]. Therefore, the strengthening effect is the best at the deformation condition of 440/0.1 s^−1^.

While the strain rate rises to 0.1 s^−1^, the degree of dissolution of insoluble particles gradually increases because of the mechanical crushing during the hot deformation, leading to a higher saturation after the solid solution. It provides a greater driving force for dispersion precipitation during aging. When the strain rate accelerates to 1 s^−1^, the dissolving degree of insoluble particles is weakened, and the precipitation driving force decreases, so the microhardness decreases. In summary, the hot deformation and subsequent strengthening of the alloy are co-excellent at the strain rate near 0.1 s^−1^. This further confirms the optimal processing area in Section 3.2.

## 5. Conclusions

(1) The Arrhenius constitutive model was developed to describe the deformation behavior of Al–10.0Zn–3.0Mg–2.8Cu alloy during hot compression. The activation energy (Q) was 160.43 kJ/mol (380–460 °C, 0.001–1 s^−1^). The optimal hot processing region of the hot processing map at 0.9 strain is 431–456 °C and 0.004–0.108 s^−1^.
(2) Under low strain rate (0.001 s^−1^), the softening mechanism includes DRV and DRX. Both CDRX and DDRX mechanisms exist in hot compression. At the low temperature (380 °C), CDRX is composed of PSN and grain boundary bulging nucleation. The PSN effect is weakened as the deformation temperature rises.
(3) The strain rate can be optimized close to 0.1 s^−1^ according to the evolution rule of the insoluble phase. The particles were refined by dislocation to assist in consuming work hardening when the strain rate increased from 0.001 to 0.1 s^−1^. The dissolving degree of insoluble particles increases gradually, which leads to the increase of microhardness of the alloy after aging.
(4) The hot processing region considering strengthening effect was optimized at 431–456 °C and the strain rate approaches 0.1 s^−1^. This will provide theoretical support for the subsequent deformation and engineering application of Al–10.0Zn–3.0Mg–2.8Cu alloy and opens up a new research idea to find the hot deformation processing for such alloys.

## Figures and Tables

**Table 1:** Chemical constituents of 7xxx aluminum alloy (wt.%).

Mg	Si	Cu	Zn	Zr	Fe	Al
3.0	0.03	2.8	10	0.15	0.06	Bal.

**Table 2:** Parameters in constitutive relationship.

β	n	A	Q (KJ/mol)	A
0.12	3.72	0.0242	160.43 (380–460 °C)	2.38 × 10^10^

**Table 3:** Calculated values of ρ_GND_ under various deformation conditions (/m^2^).

	$\dot{\varepsilon }$/S^−1^	0.001	0.01	0.1	1
T/°C					
380		5.06 × 10^15^	5.49 × 10^15^	8.18 × 10^15^	11.51 × 10^16^
440		5.81 × 10^15^	5.71 × 10^15^	8.39 × 10^15^	12.56 × 10^16^

**Table 4:** The recrystallized fraction under various deformation conditions (%).

	$\dot{\varepsilon }$/S^−1^	0.001	0.01	0.1	1
T/°C					
380		18	19.5	23.4	26.9
440		18.7	22.3	23.8	24.2

## Data Availability

Not applicable.
